# Calix[4]arene Design for Enhanced Carbon Capture via Topological Learning

**DOI:** 10.1002/chem.202503668

**Published:** 2026-02-23

**Authors:** Jeffrey A. Laub, Konstantinos D. Vogiatzis

**Affiliations:** ^1^ Department of Chemistry University of Tennessee Knoxville Tennessee USA

**Keywords:** CO_2_ Capture, Machine Learning, Molecular Design, Molecular Topology, Persistent Homology

## Abstract

Solid direct air capture (S‐DAC) technologies have the potential to remove pre‐existing CO_2_ from the atmosphere by taking advantage of hydrophobic materials that will retain CO_2_ through physisorption and separating it from the abundant water molecules. Calix[*n*]arenes are highly tunable and versatile complexes with inherent hydrophobic cavities, making them promising candidates for S‐DAC technologies. In this study, we investigated four classes of calix[4]arenes, which were functionalized symmetrically at various sites, using machine learning workflows based on molecular topology. The Topology‐Guided Carbon Capture with Calix[4]arenes (TopoC^3^) model presented here leverages DFT data from an initial molecular subset together with persistent homology–based topological descriptors to enable targeted molecular discovery. Recognizing the strong dependence of CO_2_ host‐guest interactions on molecular structure, this study identified key structural and topological features that govern adsorption performance. These insights establish clear design criteria for calix[4]arenes as molecular candidates for CO_2_ capture and provide a principled framework for further molecular engineering.

## 1 Introduction

Since the preindustrial era (1850‐1900) the average global temperature has increased over 1°C, with 2024 reporting an average global temperature increase of 1.47°C by NASA's Goddard Institute for Space Studies [1]. This increase in average global temperature has been primarily attributed to the increase in atmospheric CO_2_ with the concentration being reported as 426.46 ppm as of November 2025 [2, 3, 4]. While there are many studies focused on the removal of CO_2_ directly from flue gas in power plants [5], emissions that were not removed from point source extraction methods remain in the atmosphere. To recover past emissions of CO_2_, direct air capture (DAC) technologies are being developed and tested as promising methods to compliment point source CO_2_ capture [4, 6, 7, 8, 9].

DAC technologies have two areas of focus which include the use of liquid sorbents (L‐DAC) and solid sorbents (S‐DAC) for CO_2_ capture. While both technologies have their strengths and weaknesses, S‐DAC has an advantage due to the utilization of adsorption‐based materials, which reduce the cost of CO_2_ and sorbent material recovery [4, 6]. Indeed, it has been stated by Shi and coworkers that the development of adsorbent materials that can selectively isolate CO_2_ from ambient air with significant CO_2_ adsorption capacity will be pivotal for the future of DAC technologies [4]. To improve S‐DAC technologies, there are some key hurdles to overcome, such as, the cost of material regeneration (energy demand), the cost of the material itself, material synthesizability, material stability, material surface area and porosity, humidity, temperature, and selectivity for CO_2_ over H_2_O [4, 6, 7, 8, 9, 10]. Many of these potential hurdles can be overcome by material selection and structural optimization/design that, for example, can directly reduce the cost and improve CO_2_ selectivity from ambient air [4].

**FIGURE 1:**
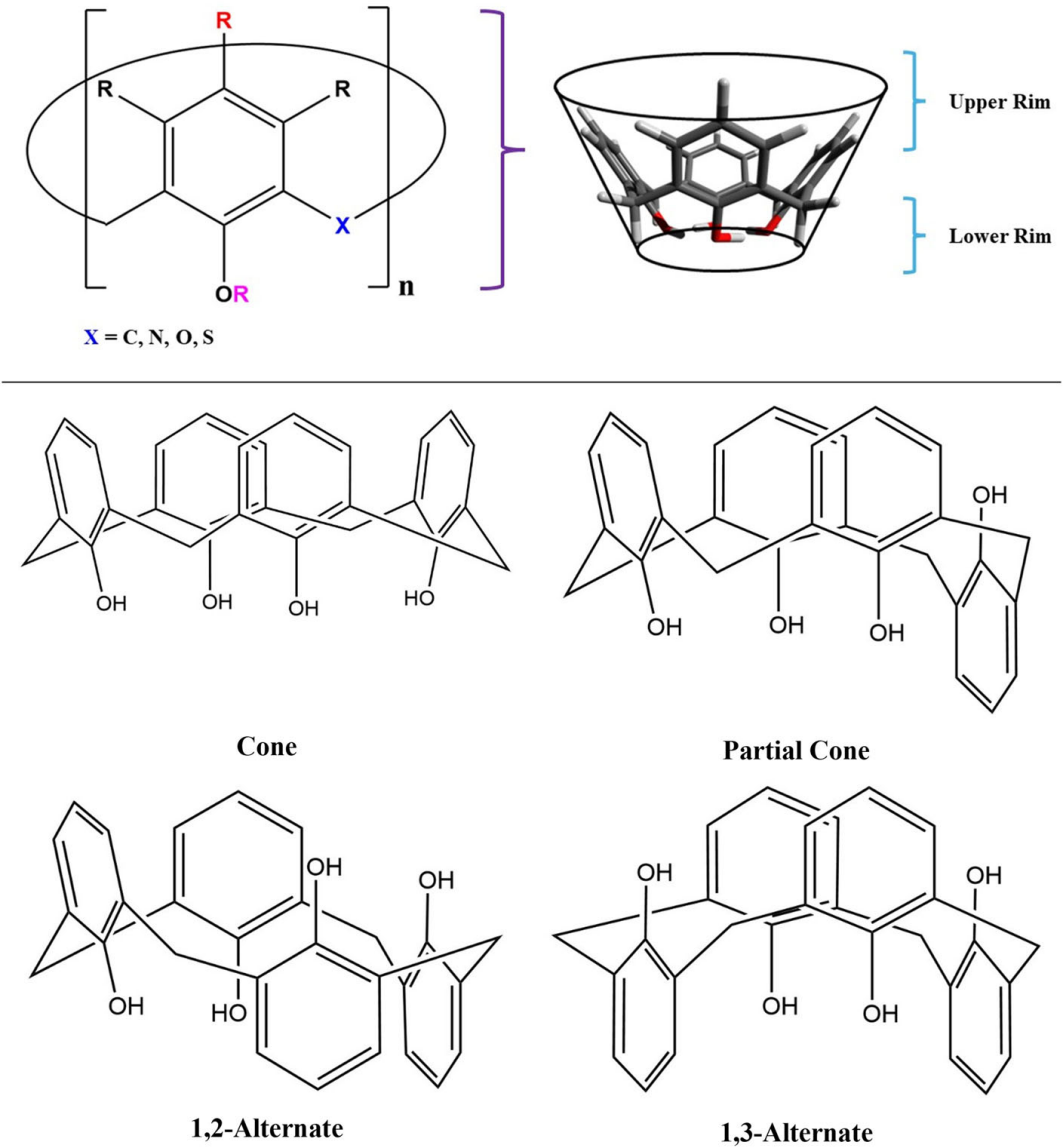
Top: (left) Calix[n]arene scaffold demonstrating the high tunability with various functional sites and (right) a visual representation of the base calix[4]arene with a chalice outline. The upper rim and lower rim are also demonstrated in the chalice representation where the lower rim also includes the bridging units. Bottom: Conformations of calix[4]arenes including the cone, partial cone, 1,2‐alternate, and 1,3‐alternate.

Calix[*n*]arenes are excellent candidates for potential implementation for DAC technologies due to their high tunability, versatility, relatively low cost for reagents and ease of synthesizability, and their ability to have host‐guest interactions with guest molecules through noncovalent interactions which reduces energy demand for material and guest molecule recovery [3, 11, 12, 13, 14, 15, 16, 17, 18]. Aptly named by Gutsche and Muthukrishnan [19], from the Greek calix meaning chalice and arene denoting aryl groups, calix[*n*]arenes consist of a hydrophobic cavity with an upper rim, a lower rim, and the “central” aryl groups as demonstrated by Figure 1 [11]. It should be noted that *n* represents the number of monomers incorporated into the cyclic oligomer, which can range from 4 as the lowest to upwards of 20 cyclic chains, where 4 through 8 are the more common cyclic oligomers [11]. The various R groups in Figure 1 are color coded to match the coupled functionalization of the calix[*n*]arene scaffold. For instance, the black *R* groups represent symmetrical functionalization of the meta position with respect to the typical hydroxyl group of the monomer, the red R demonstrates para substitution with respect to the hydroxyl group, the magenta *R* is associated with the hydroxyl group, while commonly just H, can be functionalized by substitution of the H atom, and finally, the X bridging group that is commonly a methylene linker, can indeed be N, O, or S with further functionalization when this linker is C or N. Figure 1 also demonstrates the adoptable conformations of calix[4]arenes.

Since the initial investigation involving a hemispherical calixarene (hemicarcerand) for CO_2_ capture by Cram and coworkers [20], numerous studies have investigated CO_2_ capture across a range of functionalized calix[*n*]arenes [3, 12, 13, 14, 15, 16, 17, 18, 21, 22, 23, 24, 25, 26, 27], with a few being pertinent to S‐DAC. One commonly utilized S‐DAC technology involves class 2 sorbents which are covalently tethered amine molecules to a silica substrate [6, 9, 10, 28]. Calix[4]arenes have been utilized as covalently tethered porous materials to silica gel as sorbents for medicinal purposes such as protein separations [29] and adiabatic drug adsorption [30], for stationary phases in high‐performance liquid chromatography [31], and for CO_2_ adsorption [3]. The CO_2_ adsorption study by Taghizadeh and coworkers investigated structural‐based effects on CO_2_ adsorption by linking the calixarene to silica gel by use of 2,4‐toluene diisocyanate [3]. The study reported CO_2_ enthalpy of adsorption (*Q*_st_) values for the bare silica gel (5.60 kcal·mol^−1^), calix[4]arene (5.71 kcal·mol^−1^), *p*‐*tert*‐butylcalix[4]arene (5.70 kcal·mol^−1^), and tetraaminocalix[4]arene prepared with a high amine loading (9.03 kcal·mol^−1^) and low amine loading (7.83 kcal·mol^−1^) [3]. Their results, based on the *Q*_st_ values, demonstrated that with an increase in amine groups of the calix[4]arene scaffold, the host‐guest interactions were increased, while the base calix[4]arene and *tert*‐butyl functionalized calix[4]arene performed slightly better than the bare silica gel however comparable [3]. They also tested the selectivity of these calixarenes for CO_2_ against N_2_ and CH_4_, and determined that all calixarenes exhibited a higher selectivity for CO_2_ over both gases and that the calixarene (tetraaminocalix[4]arene) with the higher amine content also had the highest selectivity for CO_2_. Unfortunately, this study did not analyze the linker effect on CO_2_ interactions for tethering the calixarene scaffolds to the silica substrate, which can cause significant changes in the interactions by varying the length of the relatively linear linker chain. In other words, the more flexible the tethered system is, the greater the chance for a reduction in CO_2_ interactions with the more favorable interaction site of the calix[4]arene to occur. Another study by Pedrini and coworkers investigated CO_2_ and CH_4_ adsorption of covalent calix[4]arene‐based frameworks [15], a type of covalent organic framework which are commonly used for CO_2_ adsorption^156‐159^ and for S‐DAC [4]. The study took advantage of the preorganization of the various calixarene monomers to synthesize the porous framework by utilizing tetrabrominated calix[4]arenes with different lower rim functionalities of the hydroxyl groups as starting materials [15]. Two of these frameworks were constructed using the *p*‐*tert*‐butylcalix[4]arene (cone conformation) and 5,11,17,23‐tetrabutyl‐25,26,27,28‐tetramethoxy‐calix[4]arene (partial cone conformation) which, when analyzed using microcalorimetry and adsorption isotherms for low coverage CO_2_ adsorption, resulted in *Q*_st_ values of 8.37 kcal·mol^−1^ and 6.21 kcal·mol^−1^, respectively [15]. Both calix[4]arenes also demonstrated selectivity for CO_2_ over N_2_ which was highlighted to be used as a possible flue gas treatment alternative [15].

While direct demonstrations of calixarene materials in DAC applications remain sparse, prior work shows that calix[4]arene derivatives, especially when functionalized and immobilized on solid supports, exhibit high CO_2_ selectivity and strong binding interactions, underscoring their potential as tunable building blocks for DAC sorbents [16, 22, 25, 32]. Calix[4]arenes are relatively inexpensive and easily synthesizable [11] and have already been demonstrated to be used in similar methods that are currently being utilized in S‐DAC technologies [3, 15]. As demonstrated by the variation in enthalpic values for CO_2_ adsorption in the aforementioned studies, by altering the structure of the calix[*n*]arenes, the strength of the noncovalent interactions varies. Custelcean and coworkers estimated that for generic solid sorbents with moderately efficient CO_2_ capture capabilities, that the enthalpy of CO_2_ sorption from ambient air should be around ‐11.95 kcal·mol^−1^ for DAC technologies [33, 34]. By exploring a large chemical space of calix[4]arene derivatives, improvement of the enthalpy of adsorption of CO_2_ can be accomplished to determine candidates for S‐DAC testing and components from the functional groups that can improve the *Q*_st_ values to be around the optimum enthalpy value predicted by Custelcean and coworkers [33, 34]. Indeed, a previous computational study investigated four functional sites of calix[4]arene with 13 different functional groups [12]. This study demonstrated that functionalization at the *para* position relative to the hydroxyl groups enhances noncovalent interactions with CO_2_, with the largest change in computed energies observed for the methoxyamine substituent [12].

In this work, we present the Topology‐Guided Carbon Capture with Calix[4]arenes (TopoC^3^), a novel machine learning (ML) workflow that utilizes quantum chemical data together with topological descriptors for the examination of a large molecular database of functionalized calix[4]arenes and their derivatives. First, a conformational search was performed to identify the most favorable structure for subsequent density functional theory (DFT) optimizations. Then, a dataset of functionalized organic oligocycles was generated using calix[4]arene, azacalix[4]arene, oxacalix[4]arene, and thiacalix[4]arene as initial molecular scaffolds. Data generation with DFT was performed on a representative subset of molecules and the resulting optimized geometries of the host complexes were encoded as topological descriptors and used to train a ML model for topology‐guided molecular discovery. Application of TopoC^3^ enabled the exploration of the full database of more than 24,000 organic oligocycles from all four molecular families. A subsequent validation step and iterative dataset augmentation further improved the accuracy of the TopoC^3^ workflow. The resulting predictions were then used to evaluate promising candidates and the identification of additional calix[4]arene design criteria that enhance CO_2_ host‐guest interactions.

## 2 Computational Details

### 2.1 Generation of the Calix[4]arene Database

**FIGURE 2:**
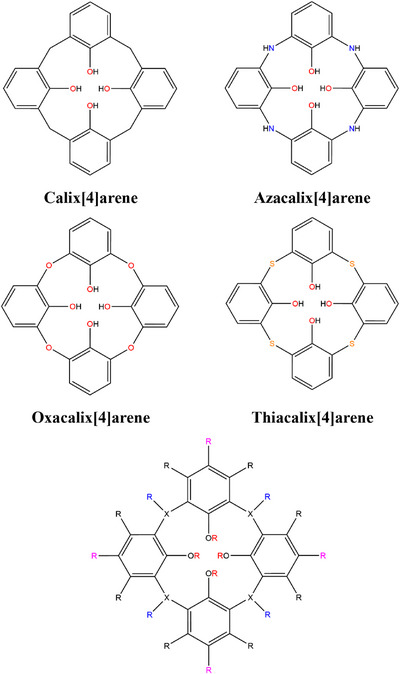
Molecular structures of the parent calix[4]arene, azacalix[4]arene, oxacalix[4]arene, and thiacalix[4]arene with colored N (blue), O (red), and S (amber) atoms. The substitution sites (color‐coded R groups) for symmetrical functionalization are shown at the bottom. The oxacalix[4]arene and thiacalix[4]arene structures do not have R site (blue) functionalization at the bridging atom.

A molecular database of organic oligocycles was generated by using four parent structures of calix[4]arene, azacalix[4]arene, oxacalix[4]arene, and thiacalix[4]arene (Figure 2). Symmetrical molecular substitutions were performed in the upper rim, lower rim, and the bridging atom for all the parent structures (no bridging atom functionalization for oxacalix[4]arene and thiacalix[4]arene). The molSimplify [35] package was used for the direct substitution of a H atom in the *R* sites of the preoptimized parent molecules in the cone conformation. The substitution involved 26 SMILES [36, 37] strings from the GDB3 database, a subspace of the GDB11 database [38, 39], containing SMILES strings consisting of C, O, N, and F atoms of one, two, and three atom strings. The SMILES strings were filtered to exclude all strings that would result in improper valency, such as the SMILES string FC, which would result in the F atom being coordinated at the *R* site and would yield an unreasonable molecular structure. This is due to the architecture of the molSimplify code that coordinates the first atom listed in the SMILES string to the classified site for substitution. The following 26 SMILES strings were used for functionalization of the four parent structures: C, N, O, F, CC, CN, CO, CF, C = C, C = O, CCC, CCN, CCO, CCF, CNC, COC, CC = C, CC = O, NC = N, NC = O, OC = O, NN = C, ON = C, C1CC1, C1CN1, and C1CO1. Symmetrical functionalization of the calix[4]arenes involved the color‐coded *R* groups in Figure 2. The magenta *R* groups represent the symmetrical functionalization at the *para* position, black represents *meta* functionalization, blue represents bridging functionalization (only applicable for calix[4]arene and azacalix[4]arene), and red represents lower rim functionalization. For example, a collection of generated structures was obtained by functionalizing the four magenta *R* groups with 26 SMILES strings, resulting in 26 unique calix[4]arene structures. It should be noted that molSimplify treated the SMILES strings O and CO as a double bonded O and a carbonyl functional group (which is already included in the SMILES string list as C = O), respectively, when it should have functionalized the calix[4]arene scaffolds as a hydroxyl group and a methanol group, respectively. Due to this occurrence, manual corrections were applied to those specific cases by adding a hydrogen atom to the functional group and reducing the bond order. We should also note that the unfunctionalized forms of each calix[4]arene family as well as the *p*‐*tert*‐butyl substituted structures were used in this study by manual addition to the database. Overall, this dataset was composed of 345 unique molecular oligocycles (97 calix[4]arenes, 101 azacalix[4]arenes, 75 oxacalix[4]arenes, and 72 thiacalix[4]arenes) and used for generating DFT data that were utilized for the training of the TopoC^3^ ML workflow.

The extended chemical space that was explored by the TopoC^3^ workflow was also generated in the same manner but utilized larger molecular substituents from a filtered GDB11 database with 4–6 atom SMILES strings for the generation of 24,214 unique molecular structures. Some SMILES strings failed to generate a complex commonly due to overlapping atoms upon functionalization of specific sites of the calix[4]arenes and were excluded. Details on data curation of the molecular dataset are provided in the Supporting Information.

### 2.2 Conformation Screening and Optimization of Calix[4]arene Host‐Guest Complexes

Given the substantial conformational flexibility of calix[4]arenes and their variants, which arise from both functional group identity and functionalization site, a systematic approach was employed to efficiently screen their conformational space. For that purpose, we applied the Conformer‐Rotamer Ensemble Sampling Tool (CREST, version 2.12) [40] with the GFN2‐xTB [41] method for structure optimization. The conformational space was screened by using the host‐guest complexes (i.e. those containing CO_2_ in the chalice) as the initial structure to screen the more favorable conformation of the oligocycle and the interacting position of CO_2_. The conformation search resulted in oligocycles adopting cone, partial cone, 1,2‐alternate, or 1,3‐alternate conformations (Figure 1), with the CO_2_ molecule located at an energetically favorable position. The lowest energy conformer determined by CREST was used for DFT calculations, where the CO_2_ molecule was removed from this conformer to perform DFT optimization on the *host* oligocycle structure.

All DFT computations in this study were performed with the ORCA 5.0.4 quantum chemistry package [42, 43, 44]. A small benchmark study (see Supporting Information) was performed to determine the optimum level of theory. The benchmark study involved a comparison between BP86 [45, 46], BLYP [45, 47], PBE [48, 49], B3LYP [47, 50], PBE0 [51], M06 [52], and *ω*B97 [53] density functionals with the def2‐SVP, def2‐TZVP, and def2‐TZVPP basis sets [54, 55]. The results demonstrated that the choice of basis set has a more pronounced effect on the ∆*H* than the choice of the density functional. Across all density functionals, the def2‐TZVP and def2‐TZVPP basis sets had similar mean absolute differences with respect to the experimental *p*‐*tert*‐butylcalix[4]arene CO_2_ *Q_st_* value (0.50 ± 0.31 kcal·mol^−1^ and 0.40 ± 0.42 kcal·mol^−1^, respectively). From this analysis, the BP86 [45, 46] density functional was chosen with the def2‐TZVP [56, 57] basis set due to the relatively low computational cost, especially for the analysis of hundreds of organic oligocycles. The def2/J auxiliary basis set, D4 [58] dispersion corrections, and the resolution of identity for the calculation of the two electron integrals were applied in all DFT computations. In addition, the Δ*H* for the *p*‐*tert*‐butylcalix[4]arene from the BP86‐D4/def2‐TZVP level of theory (‐7.96 kcal·mol^−1^) was within reasonable accuracy when compared to the experimental value of ‐8.37 kcal·mol^−1^ reported by Pedrini and coworkers [15].

Numerical harmonic frequency calculations were performed on all structures to compute the molecular enthalpy at 298.15 K. All CO_2_ adsorption enthalpies were computed using Equation (1):

(1) $$\Delta H=H_{\mathrm{Calix}+CO_{2}}-H_{\mathrm{Calix}}-H_{CO_{2}}$$

We note here that any structure that had improper structures after CREST screening or DFT optimizations (such as imaginary frequencies lower than ‐50 cm^−1^) were excluded from this study.

The DFT refined molecular dataset consisted of equilibrium host complexes obtained from the previously discussed geometry optimization methodology. This choice reflected the intended use of the surrogate model for rapid screening workflows in which optimized host‐guest interaction configurations were evaluated. We note that adsorption enthalpies are ensemble properties and may depend on finite‐temperature sampling of both host conformers and off‐equilibrium host–guest arrangements. As such, the present model may be less reliable for strongly distorted configurations or systems with multiple thermally accessible host conformations. Incorporating snapshots from molecular dynamics or perturbed geometries is an alternative route that could be an area of future exploration.

Zeroth‐order symmetry‐adapted perturbation theory (SAPT0) [59] with density fitting for the self‐consistent field method was performed with the Psi4 program package [60] for the decomposition of the energy contributions from the DFT optimized host‐guest systems. The SAPT0 analysis was considered only for the 10 highlighted calixarenes in this study (*vide infra*). The energy was decomposed to provide the contributions from the dispersion, electrostatic, exchange, and induction terms to substantiate the recommended molecular design of calix[4]arenes for improving host‐guest interactions with CO_2_. These calculations involved the jun‐cc‐pVDZ [61] basis set with the jun‐cc‐pVDZ‐JKFIT [62] auxiliary basis set as recommended by Hymel and coworkers [12] and Parker and coworkers [63] for noncovalent interactions. All SAPT0 calculations also used the superposition of atomic densities method as an initial SCF guess with an energy and density convergence threshold of 1×10^−8^.

### 2.3 Molecular Topology and Persistence Images

**FIGURE 3:**
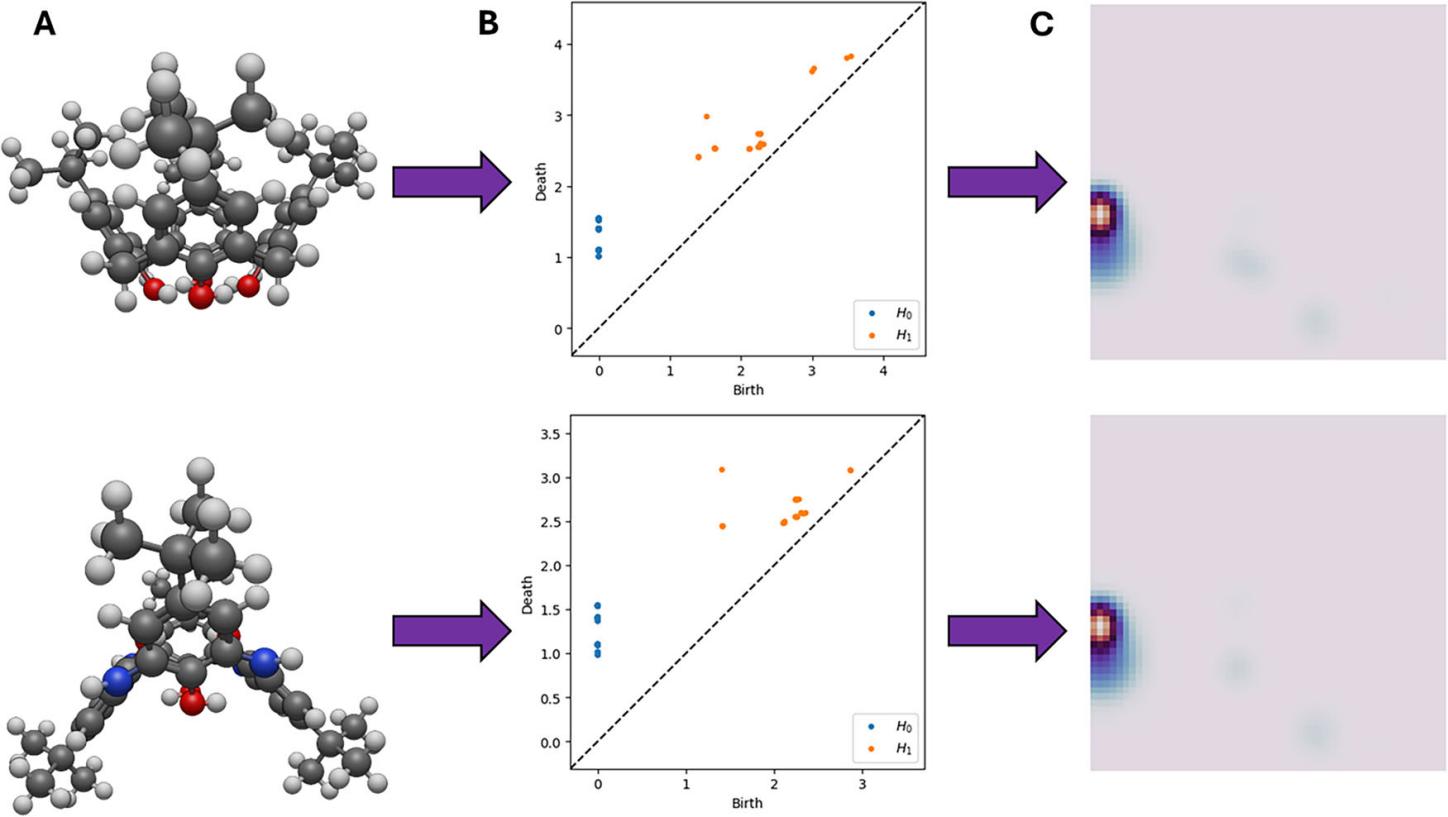
Topology‐guided descriptor flowchart that was used in this study. The Cartesian coordinates of (A) molecular structures are used for the generation of (B) persistence diagrams, which are further vectorized into (C) persistence images. The blue points in the persistence diagram represent the zeroth‐order (H_0_) homological features and the orange points are first‐order (H_1_) features. In the persistence images, the more intense the pixelation, the greater the frequency of homological features. The top row shows the molecular structure, persistence diagram, and persistence image of p‐tert‐butylcalix[4]arene, and the bottom row the same information for the p‐tert‐butylazacalix[4]arene.

Persistent homology has been recently introduced as a prominent molecular descriptor since it captures key geometric features for use in machine learning models, a methodology that is often mentioned in the literature as “topological learning” [64]. In this study, molecular topology, encoded as persistence images, were utilized by the vectorization of persistence diagrams using the deconstructed point cloud of the host calixarene cartesian coordinates with the Vietoris‐Rips filtration parameter [65]. The zeroth‐ and first‐order homological features of the molecular structures were captured across different scales by gradually increasing the radii of balls centered on each atom. The birth and death of these features are then stored in persistence diagrams (Figure 3B). From a chemical standpoint, the zeroth‐order homological features (*H*_0_) capture the connectivity (chemical bonds) between atoms, and the first‐order (*H*_1_) homological features capture the rings (or holes) formed between atoms. The persistence images were then generated from the persistence diagrams by vectorization of the homological features via calculation of the persistence and Gaussian probability density (spread) to fixed‐size descriptor images where the pixel intensity correlates to the frequency of the representative feature (Figure 3**C**) [65]. It should also be noted that for this study, the electronegativity between neighboring atoms of the zeroth‐order connectivity features was monitored and provided a variance between the components for additional information incorporated into the spread. Using fixed‐size machine‐learning descriptors that still capture the essential homological features is a major advantage, reducing computational cost relative to descriptors such as the smooth overlap of atomic positions, which are typically more expensive to compute and often require zero‐padding to enforce a fixed input size.

### 2.4 Predictability of Host‐Guest Enthalpies Using Topological Learning

The supervised ML algorithm included persistence images (PIs) [65, 66, 67, 68, 69, 70] as the molecular descriptor inputs to test the predictability of the studied calix[4]arenes. The elemental PIs descriptor, which incorporates the differences in electronegativity between neighboring atoms for the connected components and 1D homological features, was used for generating 55×55 square PIs with a standard deviation of the Gaussian kernel (spread) of 3.0, an upper boundary of the PIs of 3.5 Å, and a lower boundary of the PIs of ‐0.10 Å. With the elemental PIs of the DFT optimized host calixarene geometries without CO_2_ coupled with their respective enthalpy of adsorption value, Kernel Ridge Regression (KRR) was used as implemented in the Scikit‐learn package [71] using Python 3.13.2. KRR was selected because it is well suited to the dataset size, requires only a small number of hyperparameters for efficient screening and tuning, enables rapid model evaluation, and delivers performance comparable to more complex, hyperparameter‐rich decision tree‐based models such as random forest regression. The host calixarene structures were chosen over the host‐guest molecular supersystem for model simplicity and due to their similar behavior with respect to the host structures, since it was not desired to have the entire supersystem optimized for screening potential candidates. Instead, it was designed to only need the host calixarene complex. In regard to similar behavior with respect to the host structures, it was observed that, for example, when using the 345‐molecule dataset for training a model with the host‐guest supersystem and for the host calixarenes, the resulting statistical values were quite similar. The computed R^2^ between the training set of the two models differed only by 0.0022 in favor of the simpler model based on host calixarene structures. Similarly, the R^2^ difference between the testing set of the two models was only 0.0056, which further demonstrated that no additional information was learned from the supersystems for this study.

The KRR model utilized a 3‐fold cross validation process with a Laplacian kernel. The *α* and *γ* hyperparameters were determined using a grid search and the one‐standard‐error rule for optimizing the average R^2^ value for the testing set while preventing overfitting. This resulted in *α* and *γ* hyperparameter values of 0.27 and 0.041, respectively, and an average R^2^ score of 0.89 for the training set (230 molecules) and 0.58 for the testing set (115 molecules) across 3 cross‐validation folds. The overall average root mean square error (RMSE) of the trained model across the 3‐folds was 1.38 ± 0.11 kcal·mol^−1^ and the average mean absolute error (MAE) was 0.98 ± 0.04 kcal·mol^−1^.

Additional grid searches were performed for the *α* and *γ* hyperparameter values with each additional data inclusion into the subset (see Supporting Information section “TopoC^3^ Parity Plots” for the parity plots and statistical analysis of the individual 3 folds for the trained models with the 345‐, 356‐, and 361‐molecule datasets). The first addition included 11 structures with respective ∆*H* data and resulted in the *α* and *γ* hyperparameter values of 0.22 and 0.034, respectively, with an average R^2^ score of 0.89 for the training set (237 molecules), 0.58 for the testing set (119 molecules), and an overall average RMSE and MAE of the trained model of 1.42 ± 0.05 kcal·mol^−1^ and 1.00 ± 0.03 kcal·mol^−1^, respectively, across a 3 fold cross‐validation. The final additional data included 5 structures and resulted in the *α* and *γ* hyperparameter values of 0.10 and 0.034, respectively, with an average R^2^ score of 0.95 for the training set (240 molecules), 0.61 for the testing set (121 molecules), and an overall average RMSE and MAE of the trained model of 1.43 ± 0.05 kcal·mol^−1^ and 1.02 ± 0.05 kcal·mol^−1^, respectively, across a 3 fold cross‐validation.

## 3 Results and Discussion

### 3.1 Structural Contributions of Calix[4]arenes towards Increasing CO_2_ Enthalpy of Adsorption

**FIGURE 4:**
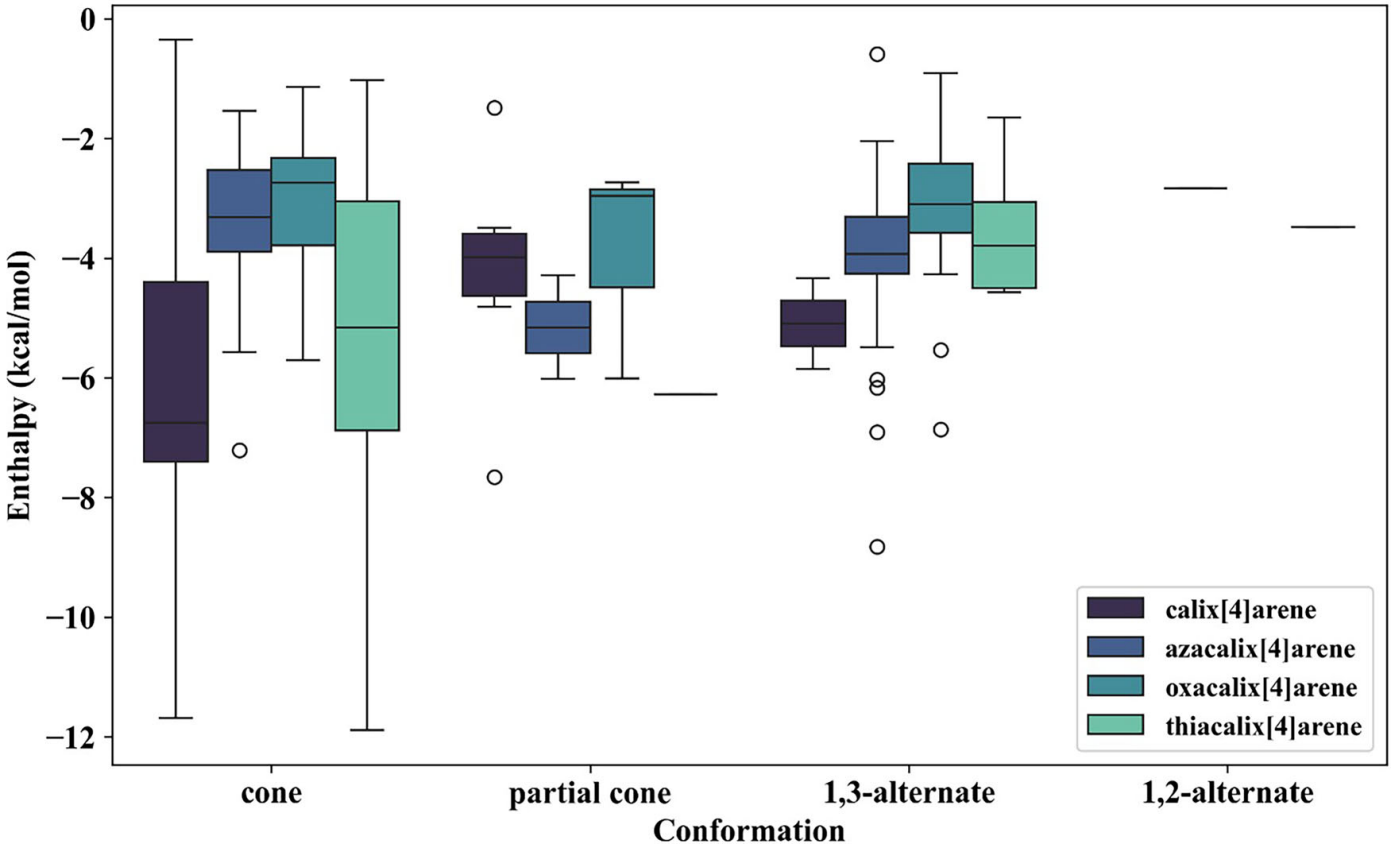
Box plots for the calix[4]arene, azacalix[4]arene, oxacalix[4]arene, and thiacalix[4]arene derivatives with respect to the most stable conformation determined from CREST, followed by DFT geometry optimization to determine the host‐guest enthalpy of CO_2_ adsorption. Boxes indicate the interquartile range (Q1–Q3) with the median shown as a horizontal line; whiskers extend to data points within 1.5× the interquartile range, and outliers are shown as circles.

The dataset of 345 molecular oligocycles was the starting point of this study. The distribution of these calix[4]arenes and their derivatives with respect to the computed CO_2_ enthalpies of adsorption is demonstrated in Figure 4. The results are also organized with respect to the most stable conformation (cone, partial cone, 1,2‐alternate, or 1,3‐alternate), as shown in separate box plots. Precisely, the cone conformation consisted of 89 calix[4]arene, 27 azacalix[4]arene, 26 oxacalix[4]arene, and 65 thiacalix[4]arene complexes; the partial cone conformation consisted of 6 calix[4]arene, 2 azacalix[4]arene, 3 oxacalix[4]arene, and 1 thiacalix[4]arene complexes; the 1,3‐alternate conformation consisted of 2 calix[4]arene, 71 azacalix[4]arene, 46 oxacalix[4]arene, and 5 thiacalix[4]arene complexes; and the 1,2‐alternate conformation consisted of 1 azacalix[4]arene and 1 thiacalix[4]arene complexes. For the calix[4]arene and thiacalix[4]arene complexes, the cone conformation was found to yield the highest enthalpies of adsorption. In contrast, azacalix[4]arenes exhibited more favorable adsorption enthalpies in the partial cone conformation, with the most favorable complex adopting a 1,3‐alternate conformation bearing a *meta*‐substituted –CH_2_CH_2_NH_2_ group. This enhanced binding may arise from weak covalent interactions between CO_2_ and the amine groups. Oxacalix[4]arenes displayed the lowest median adsorption enthalpies across all conformations examined, which were similar across cone, partial cone, and 1,3‐alternate cases. Among these, the most favorable median enthalpy was associated with the partial cone conformation, while the strongest‐binding complex appeared as an outlier in the 1,3‐alternate conformation with a *meta*‐substituted –CH_2_OH group, likely due to additional hydrogen‐bonding interactions. One final observation on the conformation behavior of these calix[4]arenes was the similarity between the calix[4]arene and thiacalix[4]arene complexes, which exhibit a preference for the cone conformation, as well as the azacalix[4]arene and oxacalix[4]arene complexes which adopted the 1,3‐alternate conformer as the most dominant conformation.

**FIGURE 5:**
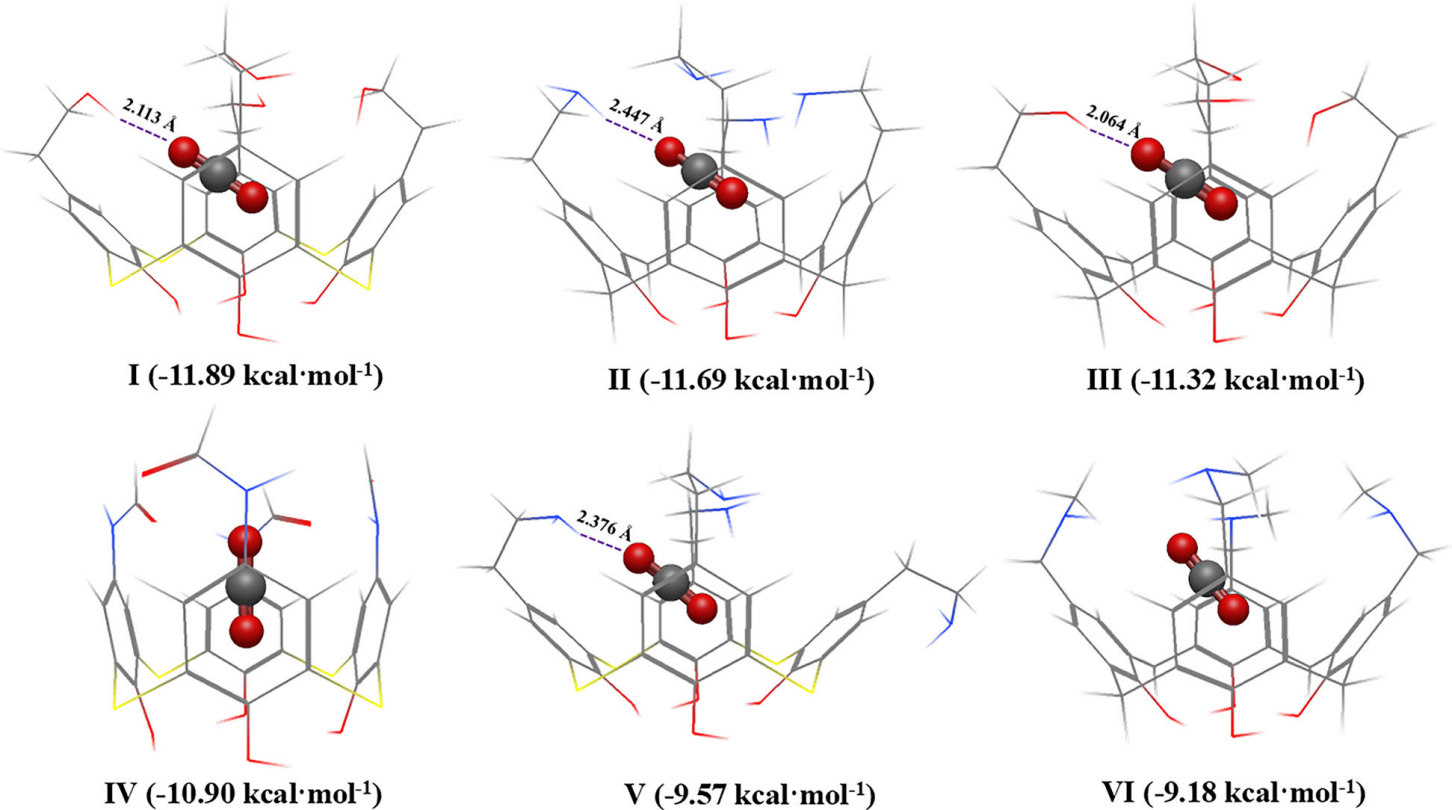
The top 6 candidates with CO_2_ enthalpy of adsorption (demonstrated in parentheses) less than ‐9 kcal·mol^−1^. The Roman numerals demonstrate the ranking starting from the most favorable host‐guest interaction (**I**) to the least favorable (**VI**). Carbon atoms are shown in grey, hydrogen atoms in white, oxygen atoms in red, nitrogen atoms in blue, and sulfur atoms in yellow. Atomic distances for hydrogen bonding interactions have been included for distances below 2.5 Å. The CO_2_ guest molecule is displayed as a ball and stick model for emphasis.

Given the favorable CO_2_ host–guest interactions observed for cone conformations in calix[4]arene and thiacalix[4]arene complexes, the top‐performing molecules among the 345 systems (Figure 5) were selected for further analysis. To evaluate the top performing calix[4]arenes, the CO_2_ enthalpies of adsorption for unsubstituted (terminated with H atoms) and *p*‐*tert*‐butyl substituted calix[4]arenes were used as reference systems, as these represent the most commonly studied calix[4]arenes. The computed ∆*H* value for unsubstituted calix[4]arene was ‐6.85 kcal·mol^−1^ (cone), ‐3.42 kcal·mol^−1^ for azacalix[4]arene (1,3‐alternate), ‐2.20 kcal·mol^−1^ for oxacalix[4]arene (1,3‐alternate), and ‐6.56 kcal·mol^−1^ for thiacalix[4]arene (cone). The ∆*H* value for symmetrical *p*‐*tert*‐butyl functionalized calix[4]arene was ‐7.97 kcal·mol^−1^ (cone, 0.41 kcal·mol^−1^ difference from experimental value [15]), ‐4.14 kcal·mol^−1^ for azacalix[4]arene (1,3‐alternate), ‐2.28 kcal·mol^−1^ for oxacalix[4]arene (1,3‐alternate), and ‐8.87 kcal·mol^−1^ for thiacalix[4]arene (cone). Across all systems examined, calix[4]arene and thiacalix[4]arene complexes in the cone conformation exhibited more favorable adsorption enthalpies than their azacalix[4]arene and oxacalix[4]arene counterparts. Accordingly, the top‐performing molecules predicted by TopoC^3^ within the 345‐member oligocycle dataset were predominantly drawn from these two families. From the top 20 candidates with enthalpy of adsorption values between ‐12.00 kcal·mol^−1^ and ‐8.00 kcal·mol^−1^, nine were from calix[4]arene, 10 were from thiacalix[4]arene, and one from azacalix[4]arene. The azacalix[4]arene‐CO_2_ variant attributed to a partially chemisorbed CO_2_ molecule at the amine site (143.5°CO_2_ bend angle and O_2_C—NH_2_ distance of 1.819 Å). Thus, calix[4]arenes and thiacalix[4]arenes are recommended for further exploration.

We will now focus on the top six candidates which were shown to all be functionalized in the *para* position. Those adopted the cone conformation and were among the calix[4]arene and thiacalix[4]arene families. Their computed enthalpies of adsorption are lower than ‐9.00 kcal·mol^−1^ and near the ‐11.95 kcal·mol^−1^ target set by Custelcean and coworkers [33, 34]. These 6 complexes are shown in Figure 5 (**I**‐**VI**); three are derivatives of calix[4]arene, and the other three are derivatives of thiacalix[4]arene. The top thiacalix[4]arene complexes include ‐CH_2_CH_2_OH (‐11.89 kcal·mol^−1^, structure **I**), ‐NHCHO (‐10.90 kcal·mol^−1^, structure **IV**), and ‐CH_2_CH_2_NH_2_ (‐9.57 kcal·mol^−1^, structure **V**) functional groups. The top calix[4]arene structures include ‐CH_2_CH_2_NH_2_ (‐11.69 kcal·mol^−1^, structure **II**), ‐CH_2_CH_2_OH (‐11.32 kcal·mol^−1^, structure **III**), and ‐CH_2_NHCH_3_ (‐9.18 kcal·mol^−1^, structure **VI**). All cases are in agreement with the study by Hymel et al. [12]. which demonstrated that substitution of the *para* site provided the more favorable host‐guest interactions with CO_2_. Two out of the three different functional groups investigated (‐CH_2_CH_2_OH and ‐CH_2_CH_2_NH_2_) also appeared among the top‐performing molecular geometries in both the present and the previous study [12]. The nucleophilic functional groups are ideal for forming a *cage‐type* molecular geometry that encapsulates the CO_2_ molecule to improve noncovalent interactions and four of the six complexes had functional groups which enhanced the host‐guest interactions via hydrogen bonding. In addition, these molecular units introduce minimal steric hindrance that does not force the rotation of a phenol linker that is observed in the less favorable calix[4]arenes.

Given the relatively limited experimental studies on CO_2_ adsorption in calixarenes, agreement between the theoretical results of this work and the available experimental observations was essential for establishing the validity of our investigation. Tsue et al. studied the crystal structure of an azacalix[4]arene functionalized with *tert*‐butyl on the *para* site for selective CO_2_ uptake and it was observed that the complex preferred the 1,3‐alternate conformation [16]. From the conformation search with CREST, the 1,3‐alternate conformation was also observed in our study for *p*‐*tert*‐butylazacalix[4]arene and that a majority (70.30%) of the azacalix[4]arene complexes preferred the 1,3‐alternate conformation. This study also stated that replacement of the *tert*‐butyl groups with more polarizable functional groups would improve the host‐guest interactions with CO_2_, which was also observed when more polar functional groups were introduced [16]. For reference, the ∆*H* value for *p*‐*tert*‐butylazacalix[4]arene from our current study was ‐4.14 kcal·mol^−1^. Substitution of *tert*‐butyl in the symmetrical *para* positions with the polar functional groups, ‐CH_2_NH_2_ (‐4.23 kcal·mol^−1^), ‐CH_2_F (‐4.26 kcal·mol^−1^), ‐CH_2_CH_2_NH_2_ (‐4.70 kcal·mol^−1^), ‐CH_2_CH_2_OH (‐4.95 kcal·mol^−1^), ‐CH_2_CH_2_F (‐4.19 kcal·mol^−1^), ‐CH_2_CHO (‐5.49 kcal·mol^−1^), ‐NHCHNH (‐4.28 kcal·mol^−1^), and ‐OCHO (‐4.49 kcal·mol^−1^), had ∆*H* values more favorable than *tert*‐butyl and were shown to all adopt the 1,3‐alternate conformation. Other polar functional groups introduced in the oligocylce backbone resulted in enthalpies of adsorption values less than the *tert*‐butyl value for azacalix[4]arene.

### 3.2 Molecular Discovery Using Topological Learning

Following the identification of promising candidates among the initial 345 complexes, ML was used to expand the search to a substantially larger chemical space. A database of more than 24,000 unoptimized calix[4]arene structures was screened using molecular topology‐based descriptors for molecular discovery. Persistence images (PIs) were used to encode molecular topology into ML‐compatible inputs derived from the DFT‐optimized host structures (i.e., in the absence of a CO_2_ guest) for all 345 calix[4]arenes considered. For clarity, we hereafter refer to this model as Topology‐guided Carbon Capture with Calix[4]arenes (TopoC^3^). Additional details regarding the construction of the PIs and the TopoC^3^ model are provided in the Computational Details section. Using the 345‐molecule subset, kernel ridge regression (KRR) was used to model a relationship between the molecular topology and the CO_2_ enthalpy of adsorption. To evaluate the predictive capabilities of TopoC^3^, a random sampling of 11 structures from the calix[4]arene and thiacalix[4]arene families (see Supporting Information) was performed with a bias towards the sampling of complexes predicted to have more favorable host‐guest interactions. The sampling acquired 3 structures with predicted enthalpies below ‐5.00 kcal·mol^−1^, 3 between ‐5.00 kcal·mol^−1^ and ‐7.00 kcal·mol^−1^, and 5 lower than ‐7.00 kcal·mol^−1^. To determine the predictive capabilities of this model, the mean absolute error (MAE) between the predicted enthalpy of adsorption and the DFT calculated values was evaluated. For the 11 oligocycles, TopoC^3^ achieved a validation MAE of 1.76 kcal·mol^−1^, which is comparable to the testing MAE of 1.38 ± 0.11 kcal·mol^−1^, reflecting reliable and consistent predictive performance. The larger deviations between predicted and DFT values occurred predominantly for structures with enthalpies below ‐8 kcal·mol^−1^, owing to the scarcity of such instances in the training dataset. Indeed, the predictions from the trained 345 calix[4]arene algorithm produced a maximum predicted value of ‐8.15 kcal·mol^−1^ and consisted of only this one instance lower than ‐8 kcal·mol^−1^. The leading candidates exhibited predicted molecular topologies that followed the trends observed in the DFT analysis of the 345 calix[4]arenes within the target enthalpy range. Of the top 20 predicted structures, all were from the calix[4]arene (14 structures) and thiacalix[4]arene (6 structures) families with all but two functionalized in the *para* site (the other two were functionalized at the bridge position).

As a next step, the DFT‐computed adsorption enthalpies of the 11 structures used in the validation step were incorporated into the original subset of the 345 molecules (dataset augmentation). The TopoC^3^ model was then retrained using this expanded dataset to improve its predicted performance. Notably, this additional set included four calix[4]arenes with DFT adsorption enthalpies below ‐8.00 kcal·mol^−1^, thereby enhancing coverage of the low‐enthalpy regime. The retrained model showed similar predictability, with an error difference of 0.04 kcal·mol^−1^. Additional dataset augmentation with 5 structures was performed bringing the total count to 361 complexes, and upon retraining of the model, similar accuracy with the previous two models was observed but with improved R^2^ values for the testing and training sets (see Supporting Information).

**FIGURE 6:**
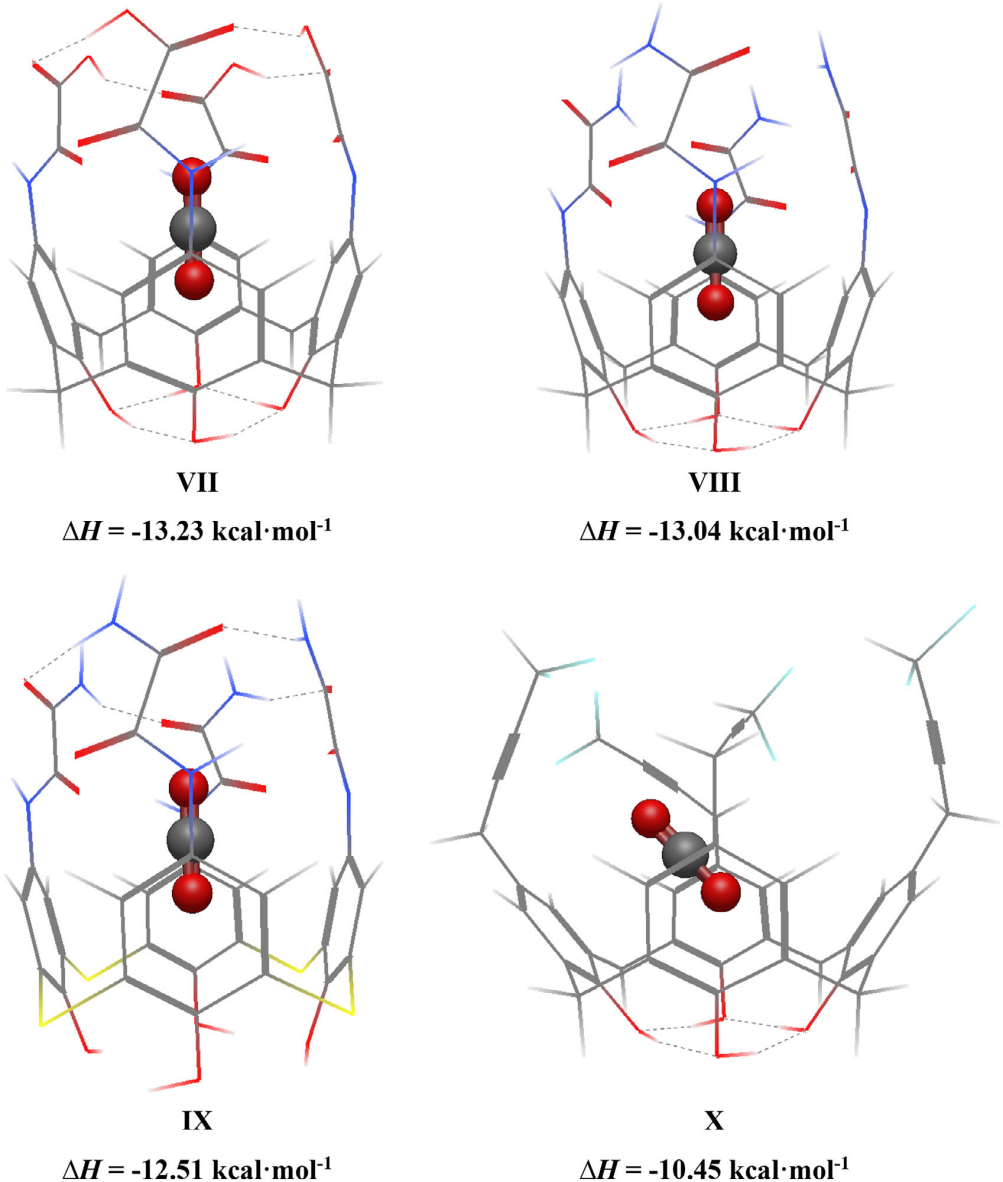
The top 4 candidates (**VII**‐**X**) from the TopoC^3^ model with their respective computed DFT CO_2_ enthalpy of adsorption values. Carbon atoms are shown in grey color, hydrogen atoms in white, oxygen atoms in red, nitrogen atoms in blue, and sulfur atoms in yellow. The CO_2_ guest molecule is displayed as a ball and stick model for emphasis.

The top 4 higher performers from the TopoC^3^ model were further analyzed and discussed (Figure 6, structures **VII‐X**). Their corresponding DFT ∆*H* values are also included in Figure 6. Structure **VII** is a calix[4]arene‐based molecule with oxamic acid symmetrically functionalized in the *para* position, structures **VIII** and **IX** have an oxamide in the *para* position (calix[4]arene and thiacalix[4]arene, respectively), while structure **X** has a fluorinated 2‐butynyl group. As is clear from Figure 6, the top potential candidates have *cage*‐like behavior where there are close contact points between the neighboring upper rim functional groups of the calix[4]arene. Another interesting observation was the difference between **VIII** and the thiacalix[4]arene variant **IX**. The difference in the DFT determined ∆*H* between the two was found to be about 0.53 kcal·mol^−1^, with the calix[4]arene outperforming thiacalix[4]arene. This difference could be due to increased stability in the calix[4]arenes due to hydrogen bonding of the lower rim which restricts the translational freedom of the guest molecule. This was also supported by the highest achieved enthalpy value of ‐13.23 kcal·mol^−1^ of structure **VII**, which also belongs to the calix[4]arene family with upper and lower rim close contact *cage*‐like features.

To provide a more thorough understanding of the electronic contributions towards the optimum host‐guest interaction energy, SAPT0 analysis was performed to decompose the total interaction into dispersion, electrostatic, exchange, and induction terms (Table 1). For all the 10 selected calixarenes (**I**‐**VI**, Figure 5 and **VII**‐**X**, Figure 6), the dispersion term was the most dominant contribution to the host‐guest interaction. The electrostatic term between structures **I**‐**III** and **V** and CO_2_ were also significantly larger than the other 6 calixarenes, which are attributed to weak hydrogen bonding interactions (see Figure 5).

**TABLE 1:** Energy decomposition of the interaction between calixarenes I‐X and CO_2_. All energies in kcal·mol^−1^.

Calixarene	*E_Dispersion_*	*E_Electrostatic_*	*E_Exchange_*	*E_Induction_*	*E_SAPT0_*
**I**	−18.61	−13.69	21.39	−3.33	−14.24
**II**	−16.84	−12.00	19.33	−2.36	−11.87
**III**	−20.27	−16.47	26.43	−4.04	−14.35
**IV**	−16.31	−6.92	12.07	−1.79	−12.96
**V**	−17.62	−12.32	20.33	−2.94	−12.54
**VI**	−15.66	−7.87	14.94	−1.82	−10.41
**VII**	−16.70	−7.97	12.03	−1.87	−14.51
**VIII**	−16.16	−7.23	10.92	−1.67	−14.14
**IX**	−14.65	−5.12	8.19	−1.28	−12.86
**X**	−17.08	−8.94	16.46	−1.94	−11.50

## 4 Conclusions

This investigation provided key structural features that can be advantageous for optimizing host‐guest interactions of calix[4]arenes and their derivatives with CO_2_ using quantum chemical data and topological learning. An initial subset of 345 functionalized calix[4]arenes was generated by introducing substituents containing one to three non‐hydrogen atoms was examined. For each complex, a conformational search was first performed to identify the preferred host geometry and CO_2_ interaction site, followed by DFT molecular optimizations to evaluate the corresponding CO_2_ enthalpies of adsorption. The DFT results were found to be consistent with available experimental observations and established theoretical models. Specifically, the calculations correctly reproduce the preference of azacalix[4]arenes for the 1,3‐alternate conformation, capture the enhancement of CO_2_ interactions upon replacement of *p*‐*tert*‐butyl groups with polar functional groups, and yielded a CO_2_ enthalpy of adsorption for *p*‐*tert*‐butylcalix[4]arene that agreed with experiment to within 0.41 kcal·mol^−^^1^.

A large database of 24,214 calix[4]arenes bearing larger functional groups containing SMILES strings of four to six non‐hydrogen atoms was explored with the Topology‐Guided Carbon Capture with Calix[4]arenes (TopoC^3^) model, a novel ML framework developed specifically for this study. The DFT data and the TopoC^3^ model agreed that symmetrical *para* functionalization of calix[4]arene and thiacalix[4]arene provided the most favorable host‐guest interactions with CO_2_ with an adopted cone conformation. The model demonstrated strong predictive performance for adsorption enthalpies within the ‐8 to 0 kcal·mol^−1^ range, which can be attributed to the higher density of training data within this region. While deviations were observed for more strongly binding candidates, the underlying molecular topology of the highest‐ranking structures was nevertheless captured correctly, enabling reliable identification of top candidates.

Lastly, the top candidates from the TopoC^3^ model, were analyzed and resulted in the discovery of the best performing calix[4]arene from this study with a ∆*H* value of ‐13.23 kcal·mol^−1^. Our study also elucidated the key molecular features that would provide potential candidates for improving CO_2_ host‐guest interactions. These molecular features involved symmetrical functionalization of the *para* site with functional groups that promote hydrogen bonding with neighboring functional groups to create a caged‐type complex with the CO_2_ molecule entrapped in the chalice. It was also found that the calix[4]arene complexes would provide improved host‐guest interactions over thiacalix[4]arene due to stronger hydrogen bonding of the lower rim for the calix[4]arene structures. The recommended design criteria for enhancing host–guest interactions were further evaluated using SAPT0 energy decomposition of the highlighted candidates, which supported our conclusions. The top 3 candidates from this study provided ∆*H* values of ‐13.23 kcal·mol^−1^, ‐13.04 kcal·mol^−1^, and ‐12.51 kcal·mol^−1^, each ower than the targeted value of ‐11.95 kcal·mol^−1^ of Custelcean and coworkers [33, 34].

## Conflicts of Interest

The authors declare no conflicts of interest.

## Supporting information




**Supporting Information File 1**: Density functional and basis set effects on the CO_2_ enthalpy of adsorption for *p*‐*tert*‐butylcalix[4]arene, parity plots from the machine learning algorithms, structural artifacts within the 24,214 calix[4]arene database, database usage reasoning. The code and all *xyz* coordinates from all optimized geometries can be found at: https://github.com/Jeffrey‐107/TopoC3.
