# Supplementary material for: Calix[4]arene Design for Enhanced Carbon Capture via Topological Learning
Source: Chemistry. 2026 Feb 23;32(18):e03668. doi: 10.1002/chem.202503668 (PMC13174912; doi:10.1002/chem.202503668)
Supplement: Supplementary file 1 — Supporting Information File 1: Density functional and basis set effects on the CO2 enthalpy of adsorption for p‐tert‐butylcalix[4]arene, parity plots from the machine learning algorithms, structural artifacts within the 24,214 calix[4]arene database, database usage reasoning. The code and all xyz coordinates from all optimized geometries can be found at: https://github.com/Jeffrey‐107/TopoC3. [file CHEM-32-e03668-s001.pdf]

Supporting information for

Calix[4]arene Design for Enhanced Carbon Capture via Topological Learning

Jeffrey A. Laub and Konstantinos D. Vogiatzis*

Department of Chemistry, University of Tennessee, Knoxville, Tennessee 37996-1600, USA

*Corresponding Author: kvogiatz@utk.edu

Table of Contents

Density Functional and Basis Set Effect 2

TopoC^3 Parity Plots 4

Structural Artifacts 7

Database Usage Reasoning 8

Structural Comparison Between Top Performers 13

References 15

Density Functional and Basis Set Effect

Before analyzing the functionalization of calix[4]arenes that increase the host-guest interaction with CO_2, a discussion on the choice of density functional and basis set is needed. To do so, the Q_{st} value of *p-tert*-butylcalix[4]arene covalent calixarene framework determined by Pedrini and coworkers[1] was used since this calixarene (monomer) has been well studied for CO_2 host-guest interactions. Since Pedrini and coworkers synthesized their frameworks with spatial preorganization with respect to the calixarene monomer, the *p*-tert-butylcalix[4]arene would be expected to have similar behavior to the crystal structure. Udachin and coworkers[2] determined that at ambient temperatures and low pressure, the host:guest interaction ratio would be 1:1 which would be around the expected conditions during application of S-DAC technologies. Using the 1:1 *p-tert*-butylcalix[4]arene:CO_2 ratio for calculation of the CO_2 inclusion enthalpy, Table S1 demonstrates the density functional and basis set effects with the targeted Q_{st} value of -8.37 kcal·mol^{-1}. We found that the basis set has greater weight on the inclusion enthalpy and that use of triple ζ basis sets provide higher accuracy. Statistical analysis across the density functionals for the different basis sets helped reinforce the choice of the level of theory. The def2-SVP basis set absolute average with the standard deviation across the density functionals was 3.97 ± 0.89 kcal·mol^{-1}, def2-TZVP was 0.50 ± 0.31 kcal·mol^{-1}, and def2-TZVPP was 0.40 ± 0.42 kcal·mol^{-1}. In terms of accuracy, the B3LYP-D4/def2-TZVPP level of theory provided the best agreement with the experimental Q_{st} value with a difference of approximately 0.02 kcal·mol^{-1}. However, using DFT with a triple ζ basis set drastically increases the amount of time required for calculation especially for these calix[4]arene molecules. Since we wished to screen many different calix[4]arene molecules, we chose to use a less accurate but significantly quicker level of theory and make note of the inherited error.

Table S1. Density Functional and Basis Set Dependent CO_2 Inclusion Enthalpy with Absolute Divergence from Experimental Values in Parentheses. All Values are in kcal·mol^{-1}.

Density Functional	def2-SVP[3, 4]	def2-TZVP[3, 4]	def2-TZVPP[3, 4]
BP86[5, 6]	-12.01 (3.64)	-7.96 (0.41)	-7.92 (0.45)
B3LYP[7, 8]	-13.12 (4.75)	-9.28 (0.91)	-8.35 (0.02)
BLYP[5, 7]	-13.68 (5.31)	-8.02 (0.35)	-8.44 (0.07)
PBE0[9]	-12.71 (4.34)	-7.47 (0.90)	-8.08 (0.29)
PBE[10, 11]	-11.77 (3.40)	-7.78 (0.59)	-7.13 (1.24)
M06[12]	-11.06 (2.69)	-8.19 (0.18)	-7.75 (0.62)
ωB97[13]	-12.02 (3.65)	-8.20 (0.17)	-8.23 (0.14)

TopoC^3 Parity Plots

<u>345 Calix[4]arenes</u>

Three-fold cross-validation was utilized for TopoC^3. The training set consisted of 230 molecules of the 345 molecule dataset and the testing set consisted of 115 molecules for each fold.

$\alpha = 0.26827$

$\gamma = 0.0409492$

Fold 1

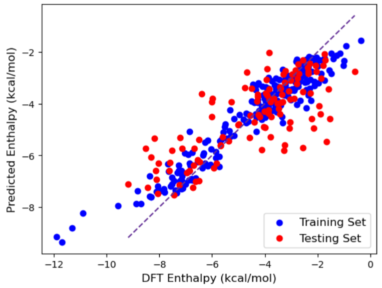

RMSE: 1.28 kcal·mol^{-1}

MAE: 0.94 kcal·mol^{-1}

Train R^2: 0.896

Test R^2: 0.587

Fold 2

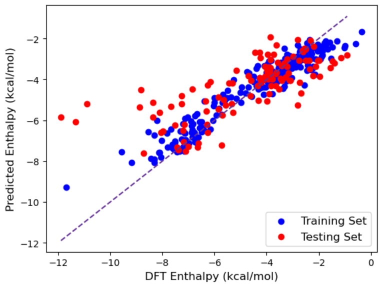

RMSE: 1.53 kcal·mol^{-1}

MAE: 1.03 kcal·mol^{-1}

Train R^2: 0.894

Test R^2: 0.518

Fold 3

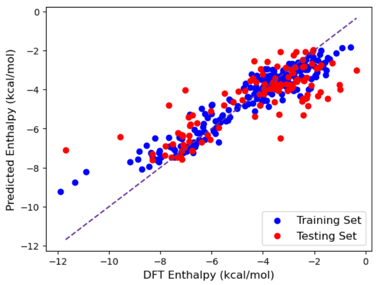

RMSE: 1.33 kcal·mol^{-1}

MAE: 0.97 kcal·mol^{-1}

Train R^2: 0.879

Test R^2: 0.625

Average across all 3 folds

RMSE: 1.38 ± 0.11 kcal·mol^{-1}

MAE: 0.98 ± 0.04 kcal·mol^{-1}

Model score: 0.733

Train R^2: 0.890

Test R^2: 0.577

356 Calix[4]arenes

α = 0.2223

γ = 0.0339322

Training set: 237

Testing set: 119

Fold 1

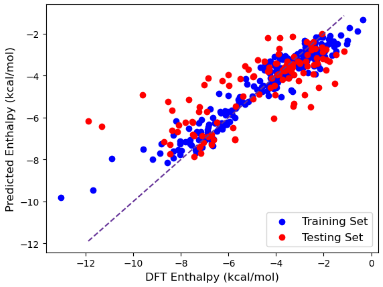

RMSE: 1.46 kcal·mol^{-1}

MAE: 1.04 kcal·mol^{-1}

Train R^2: 0.894

Test R^2: 0.576

Fold 2

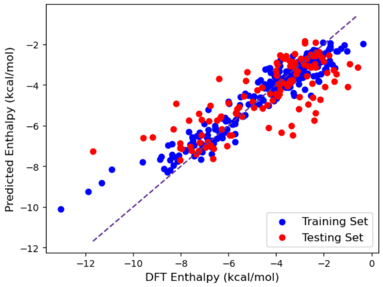

RMSE: 1.35 kcal·mol^{-1}

MAE: 0.99 kcal·mol^{-1}

Train R^2: 0.888

Test R^2: 0.603

Fold 3

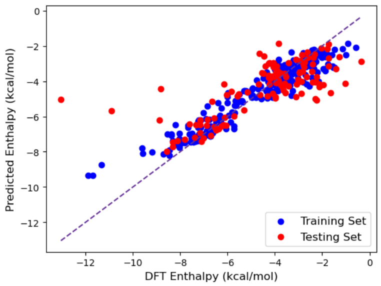

RMSE: 1.46 kcal·mol^{-1}

MAE: 0.96 kcal·mol^{-1}

Train R^2: 0.884

Test R^2: 0.547

Average across all 3 folds

RMSE: 1.42 ± 0.05 kcal·mol^{-1}

MAE: 1.00 ± 0.03 kcal·mol^{-1}

Model score: 0.732

Train R^2: 0.889

Test R^2: 0.575

361 Calix[4]arenes

α = 0.104811

γ = 0.0339322

Training set: 240

Testing set: 121

Fold 1

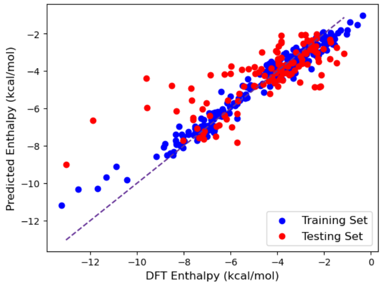

RMSE: 1.37 kcal·mol^{-1}

MAE: 0.94 kcal·mol^{-1}

Train R^2: 0.951

Test R^2: 0.592

Fold 2

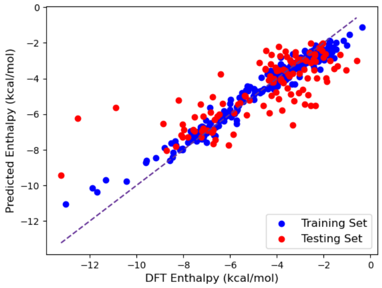

RMSE: 1.46 kcal·mol^{-1}

MAE: 1.03 kcal·mol^{-1}

Train R^2: 0.952

Test R^2: 0.608

Fold 3

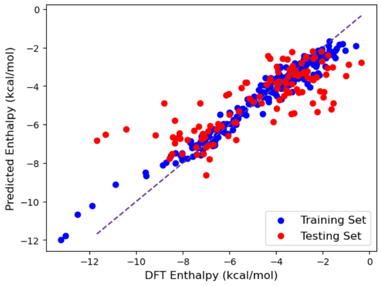

RMSE: 1.47 kcal·mol^{-1}

MAE: 1.07 kcal·mol^{-1}

Train R^2: 0.947

Test R^2: 0.619

Average across all 3 folds

RMSE: 1.43 ± 0.05 kcal·mol^{-1}

MAE: 1.02 ± 0.05 kcal·mol^{-1}

Model score: 0.778

Train R^2: 0.950

Test R^2: 0.606

Structural Artifacts

Within the original 24214 calix[4]arene database, there are possible artifacts that may need to be slightly manually adjusted before performing a conformation search with CREST. These artifacts arise by the functionalization of functional groups in *R* sites that have a greater possibility of steric hindrance. For example, **Figure S1** demonstrates the possible structural artifacts that could be encountered within the database. As demonstrated H atoms on neighboring functional groups are in close proximity to both functional groups and would need to be slightly adjusted to perform a conformation search. While these instances are not common, it needed to be addressed and does not have much of an effect on the outcomes of this work especially since these instances are observed for the calix[4]arenes that are not predicted to be within the top performing candidates to increase CO_2 host-guest interactions (*para* substituted calix[4]arene and thiacalix[4]arene). One other artifact that could be found involves the bridging of an O atom to the calix[4]arene and functional group in a linear coordination, whereas it would need to be in a bent coordination.

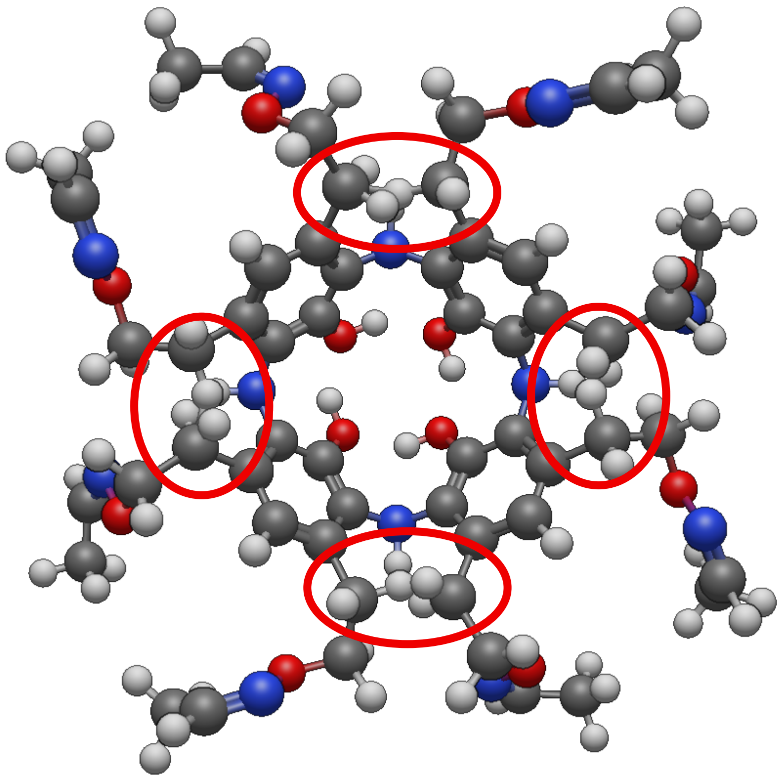

Figure S1. Example of possible structural artifacts that could be present in the calix[4]arene database. The red circles indicate H atoms that are close to two C atoms of neighboring functional groups.

Database Usage Reasoning

Typically, in machine learning applications, the database that is being used for making predictions is either optimized or organized using the same level of theory as the structures being used to train the algorithm. However, since the proper topological features of the calix[4]arenes is of great importance for our study and were used for training of the machine learning algorithm, it was imperative to determine an approximate difference between the predictions for using a database of unoptimized structures against the DFT optimized structures. **Figure S2** demonstrates the DFT calculated ΔH values (red) compared to the predicted enthalpy from the unoptimized/unaltered generated structures (black) and the DFT optimized structures (grey) using TopoC[3] with 345 molecules on the 11 calix[4]arene and thiacalix[4]arene structures from the random search (**Figure S3**).

It was observed that when using the unoptimized structures for making predictions, that the mean absolute error (MAE) between the DFT and predicted values was 1.76 kcal·mol^{-1} and the optimized structures resulted in a difference from the DFT values of 1.66 kcal·mol^{-1}. This analysis demonstrated a similar prediction in the sampling as a whole, and that, while the DFT optimized structures would perform slightly better, this was not the case for the structures with DFT values lower than -8 kcal·mol^{-1}. For the four structures with values lower than -8 kcal·mol^{-1} of the 11, the MAE in predicted values with the unoptimized versus optimized structures was 2.19 kcal·mol^{-1} and 2.77 kcal·mol^{-1}, respectively. This demonstrated that, while overall the DFT optimized structures provided a lower MAE, the fit was weighted with an emphasis on the bulk of the predicted values in the higher enthalpy values (between -8 and 0 kcal·mol^{-1}) than when using unoptimized structures from the database. This is also reinforced by the optimized structures have a MAE between the predicted and DFT enthalpy values of 0.74 kcal·mol^{-1}, while the unoptimized structures was 1.40 kcal·mol^{-1}. Thus, using only the unoptimized calix[4]arenes from the database containing over 24000 structures was viable, would remove the need for high computational cost for optimization of these structures before usage, and would be a slightly better predictor for potential candidates for increased CO_2 host-guest interactions.

One more important point to mention is that the generation of all the calix[4]arenes within the database were generated in an initial cone configuration. Upon performing a conformation search with CREST, the conformation either stayed a cone conformation or adopted a partial cone,

1,2-alternate, or 1,3-alternate conformation. So, while this would indeed play a significant role in the predictions being performed with the machine learning algorithm on the unoptimized calix[4]arenes, it is negligible with the aim of this study. This is due to the observations from the DFT study of the initial 345 calix[4]arenes which demonstrated that the cone conformation with calix[4]arene and thiacalix[4]arene would provide potential candidates within the targeted enthalpy of adsorption range as opposed to all other conformations and families. Thus, already having a database where the molecules are in the cone conformation, while not the optimum conformation for some functional groups and functionalized sites, would still be expected to produce the top potential candidates of interest due to their significantly more favorable CO_2 host-guest enthalpy of adsorption values.

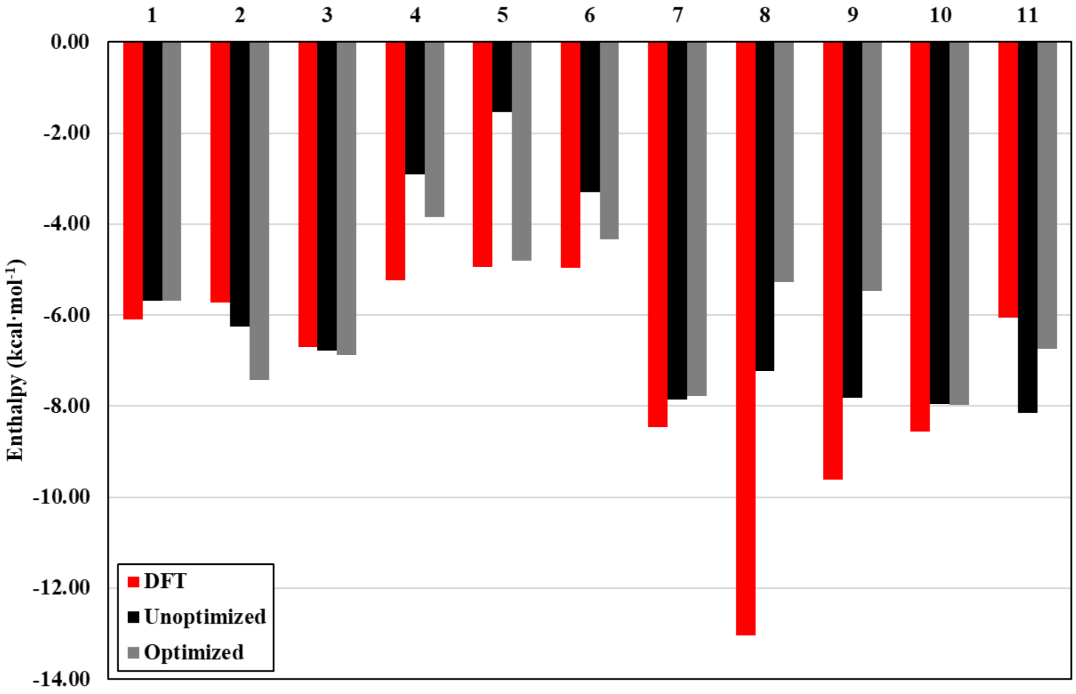

Figure S2. Comparisons between the DFT Δ*H* values of the calix[4]arenes (red) chosen from the random sampling of the database. The predictions using the calix[4]arenes directly from the database as PIs are represented by black and are labelled Unoptimized. The predictions using the DFT optimized calix[4]arenes structures as PIs are represented in grey and labeled Optimized. The *x*-axis represents the calix[4]arene number and the *y*-axis is the CO_2 enthalpy of adsorption value in kcal·mol^{-1}.

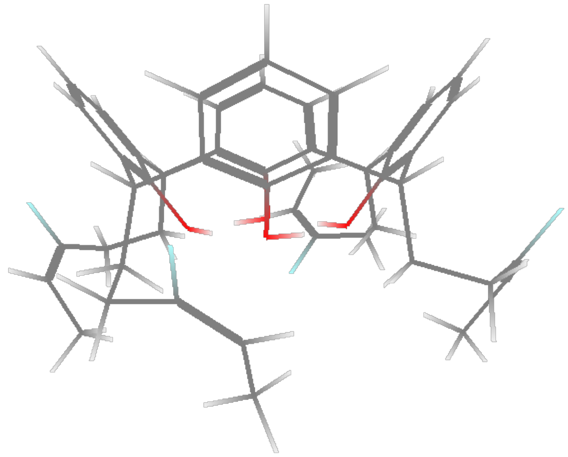

1

ΔH = -6.11 kcal·mol^{-1}

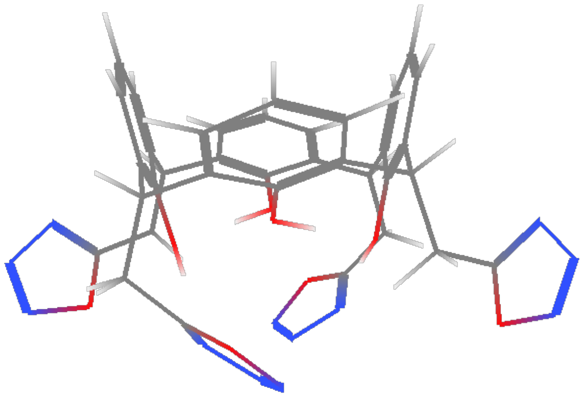

2

ΔH = -5.73 kcal·mol^{-1}

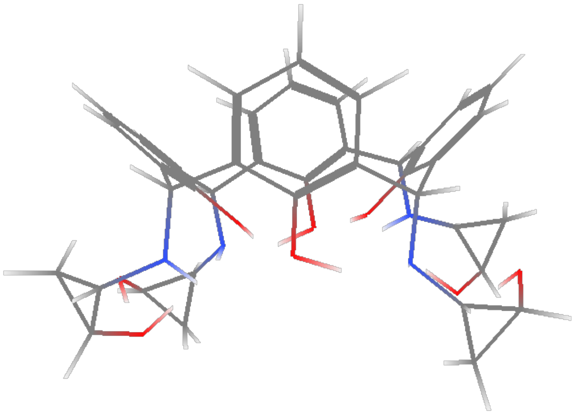

3

ΔH = -6.70 kcal·mol^{-1}

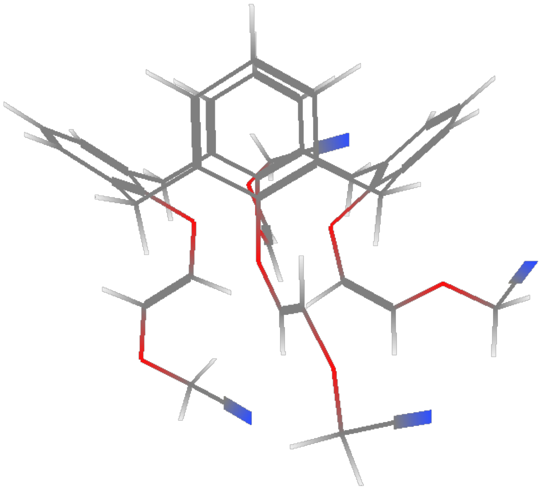

4

ΔH = -5.23 kcal·mol^{-1}

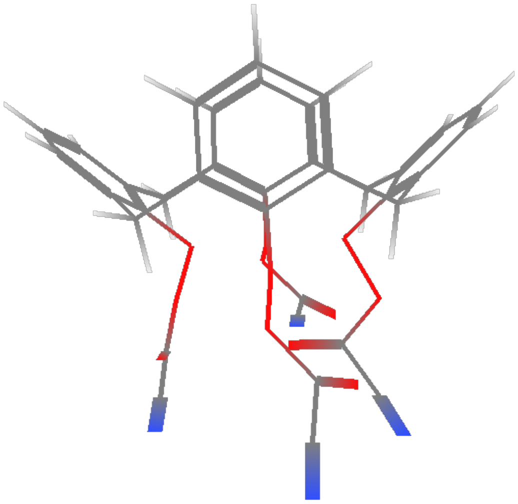

5

Δ*H* = -4.94 kcal·mol^{-1}

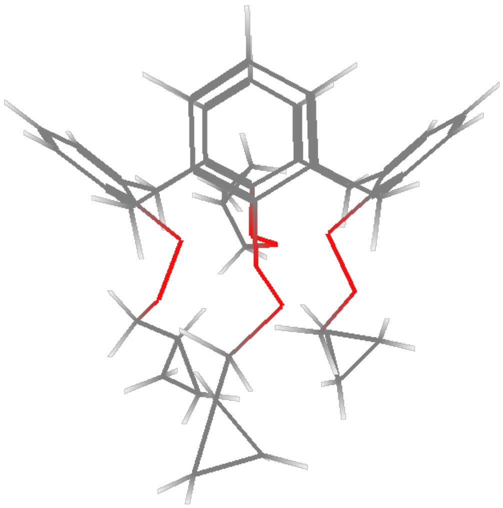

6

Δ*H* = -4.97 kcal·mol^{-1}

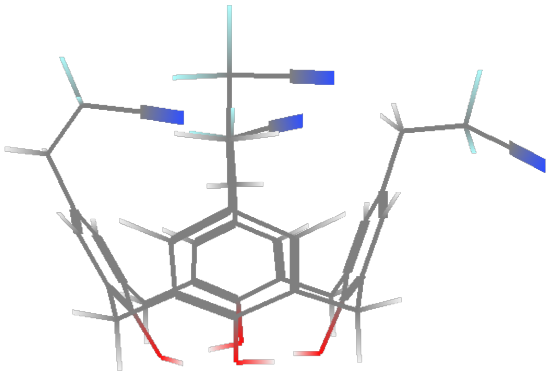

7

Δ*H* = -8.46 kcal·mol^{-1}

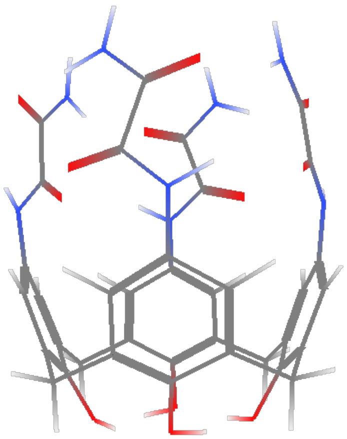

8

Δ*H* = -13.04 kcal·mol^{-1}

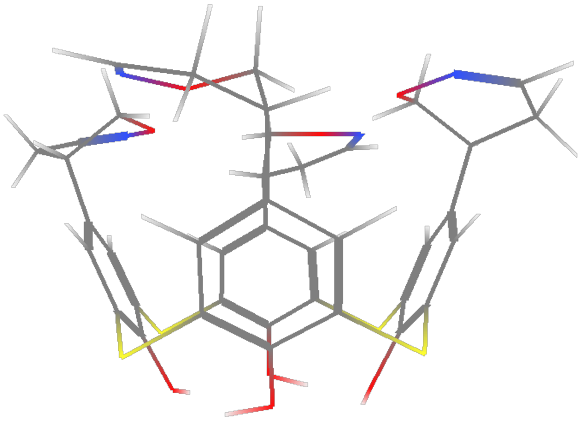

9

Δ*H* = -9.61 kcal·mol^{-1}

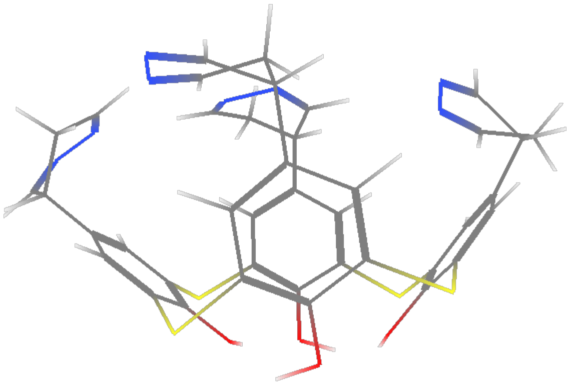

10

Δ*H* = -8.57 kcal·mol^{-1}

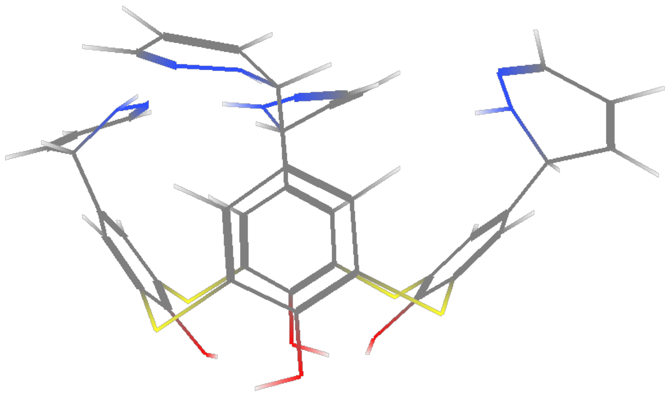

11

ΔH = -6.06 kcal·mol^{-1}

Figure S3. The 11 random structures from the calix[4]arene and thiacalix[4]arene families chosen for evaluating the predictive performance of the TopoC^3 for optimized and unoptimized structures. Each structure is numbered with their respective label and the DFT determined CO_2 enthalpy of adsorption. White atoms are H, grey are C, red is O, blue is N, and yellow is S.

Structural Comparison Between Top Performers

A short discussion on the structural differences between structures VII, XI, and XII is provided here (Figure S4). With the visual inspection of these three structures, it becomes apparent that due to the bending of the bridging S atoms (average bridging angle of 97.4°) which, consequently, increased the distances between the hydroxyl groups of the lower rim (average H---O distance of 2.603 Å), making structure **XII** more rectangular in geometry than the more capsulated cylindered-shape of **VII** and **XI**, which can be an attribute to a more favorable host-guest enthalpy.

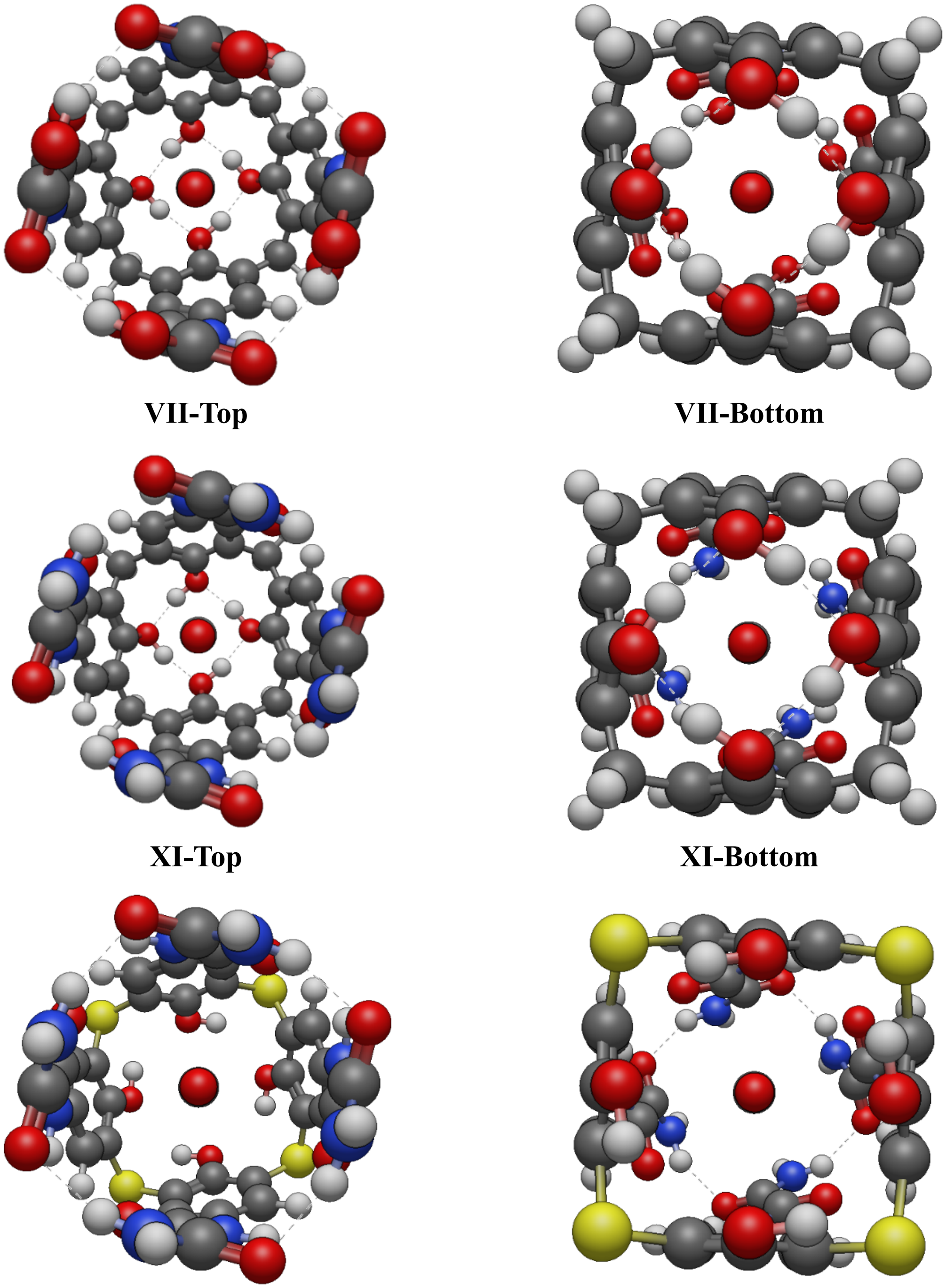

Figure S4. Top and Bottom view of **VII**, **XI**, and **XII** with the CO_2 guest molecule. The left column demonstrates the view of the host-guest model from the upper rim, while the right column demonstrates the view from the lower rim. Carbon atoms are shown in grey color, hydrogen atoms in white, oxygen atoms in red, nitrogen atoms in blue, and sulfur atoms in yellow.

References

(1) Pedrini, A.; Perego, J.; Bracco, S.; Bezuidenhout, C. X.; Sozzani, P.; Comotti, A. Calixarene-based porous 3D polymers and copolymers with high capacity and binding energy for CO_2, CH_4, and Xe capture. *J. Mater. Chem A* **2021**, *9*, 27353-27360.
(2) Udachin, K. A.; Moudrakovski, I. L.; Enright, G. D.; Ratcliffe, C. I.; Ripmeester, J. A. Loading-dependent structures of CO_2 in the flexible molecular van der Waals host *p-tert*-butylcalix[4]arene with 1:1 and 2:1 guest-host stoichiometries. *Phys. Chem. Chem. Phys.* **2008**, *10*, 4636-4643.
(3) Weigend, F.; Ahlrichs, R. Balanced basis sets of split valence, triple zeta valence and quadruple zeta valence quality for H to Rn: Design and assessment of accuracy. *Phys. Chem. Chem. Phys.* **2005**, *7*, 3297-3305.
(4) Weigend, F. Accurate Coulomb-fitting basis sets for H to Rn. *Phys. Chem. Chem. Phys.* **2006**, *8*, 1057-1065.
(5) Becke, A. D. Density-Functional Exchange-Energy Approximation with Correct Asymptotic Behavior. *Phys. Rev. A: At., Mol., Opt. Phys.* **1988**, *38*, 3098-3100.
(6) Perdew, J. P. Density-functional approximation for the correlation energy of the inhomogeneous electron gas. *Phys. Rev. B: Condes. Matter Mater. Phys.* **1986**, *33*, 8822-8824.
(7) Lee, C.; Yang, W.; Parr, R. G. Development of the Colle-Salvetti correlation-energy formula into a functional of the electron density. *Phys. Rev. B: Condes. Matter Mater. Phys.* **1988**, *37*, 785-789.
(8) Becke, A. D. A new mixing of Hartree-Fock and local density-functional theories. *J. Chem. Phys.* **1993**, *98*, 1372-1377.
(9) Adamo, C.; Barone, V. Toward reliable density functional methods without adjustable parameters: The PBE0 model. *J. Chem. Phys.* **1999**, *110*, 6158-6170.
(10) Perdew, J. P.; Burke, K.; Ernzerhof, M. Generalized Gradient Approximation Made Simple. *Phys. Rev. Lett.* **1996**, *77*, 3865.
(11) Perdew, J. P.; Burke, K.; Ernzerhof, M. Errata: Generalized gradient approximation made simple. *Phys. Rev. Lett.* **1997**, *78*, 1396.
(12) Zhao, Y.; Truhlar, D. G. The M06 suite of density functionals for main group thermochemistry, thermochemical kinetics, noncovalent interactions, excited states, and transition elements: Two new functionals and systematic testing of four M06-class functionals and 12 other functionals. *Theor. Chem. Acc,* **2008**, *120*, 215-241.
(13) Chai, J.-D.; Head-Gordon, M. Systematic optimization of long-range corrected hybrid density functionals. *J. Chem. Phys.* **2008**, *128*, 084106.
